# *Morchella* Effectively Removes Microcystins Produced by *Microcystis aeruginosa*

**DOI:** 10.1264/jsme2.ME23101

**Published:** 2024-05-18

**Authors:** Xinchao Meng, Meihan Ban, Zhaoyang Wu, Lilong Huang, Zicheng Wang, Yunqing Cheng

**Affiliations:** 1 Jilin Provincial Key Laboratory of Plant Resource Science and Green Production, Jilin Normal University, Siping 136000, China; 2 Department of Microbiology, Oregon State University, Corvallis, OR 97331, United States

**Keywords:** *Microcystis aeruginosa*, microcystin, *Morchella*, removal effect, MC-LR, MC-RR

## Abstract

Microcystins (MCs) produced by *Microcystis aeruginosa* are harmful to animal and human health, and there is currently no effective method for their removal. Therefore, the development of biological approaches that inhibit cyanobacteria and remove MCs is needed. We identified strain MB1, confirmed as *Morchella*, using morphological and mole­cular evolution methods. To assess the impact of strain MB1 on *M. aeruginosa*, we conducted an experiment in which we inoculated *M. aeruginosa* with *Morchella* strain MB1. After their co-cultivation for 4‍ ‍d, the inoculation with 0.9696‍ ‍g MB1 completely inhibited and removed *M. aeruginosa* while concurrently removing up to 95% of the MC content. Moreover, within 3‍ ‍d of their co-cultivation, MB1 removed more than 50% of nitrogen and phosphorus from the *M. aeruginosa* solution. Therefore, the development of effective biological techniques for MC removal is paramount in safeguarding both the environment and human well-being. We herein successfully isolated MB1 from its natural habitat. This strain effectively inhibited and removed *M. aeruginosa* and also reduced the content of nitrogen and phosphorus in the *M. aeruginosa* solution. Most importantly, it exhibited a robust capability to eliminate MCs. The present results offer a new method and technical reference for mitigating harmful algal blooms.

Cyanobacterial toxins are metabolites released by cyanobacteria upon cell death or lysis. Among these toxins, microcystins (MCs) are the most widely distributed and toxic (Campos and Vasconcelos, 2010; Neilan *et al.*, 2012). More than 300 MC isomers have been identified to date (Bouaicha *et al.*, 2019; Du *et al.*, 2019; Massey and Yang, 2020). MC-LR and MC-RR are relatively common and highly toxic compounds; MC-LR contains two variable amino acids, leucine (L) and arginine (R), while MC-RR contains two variable amino acids of arginine (R) (Hu *et al.*, 2017; Massey and Yang, 2020). MCs are monocyclic heptapeptide hepatotoxins that remain stable, even under harsh conditions, such as high temperatures, extreme pH, and sunlight (de la Cruz *et al.*, 2011). The inherent structural stability of MCs leads to their slow degradation in aqueous environments (He *et al.*, 2022).

MCs may reduce water quality, affect the regulation of cell protein phosphorylation, promote apoptosis (Huo *et al.*, 2021), and lead to cellular destruction and cytoskeletal damage (Zhu *et al.*, 2022). Their toxicity extends to humans and animals (Massey *et al.*, 2018; Cao *et al.*, 2019; Alosman *et al.*, 2021). Prolonged and frequent exposure to low concentrations of MCs may eventually lead to uncontrolled cell proliferation in the human body, promote the occurrence and development of tumors, and lead to primary liver cancer (Drobac *et al.*, 2013; Lee *et al.*, 2017; Woolbright *et al.*, 2017). Physical methods and chemical reactions do not offer economical or effective solutions for MC removal (Li *et al.*, 2017; Duan *et al.*, 2018). In contrast, microbial methods are widely regarded for their cost-effectiveness, environmental friendliness, and ecological restoration capabilities (Neilan *et al.*, 2012; Dziga *et al.*, 2013; Teng *et al.*, 2023). However, most MC-degrading bacteria cannot completely eliminate MCs (Massey and Yang, 2020; Zhan and Hong, 2022). Therefore, it is imperative to develop efficient biological approaches for MC removal in order to address water resource pollution and safeguard human health.

In the present study, we identified a *Morchella* strain (MB1) in the field that effectively inhibited and eliminated both *M. aeruginosa* cells and MCs. Additionally, strain MB1 exhibited the capacity to remove nitrogen and phosphorus in the *M. aeruginosa* solution tested. The present results provide evidence to support the effective management of *M. aeruginosa*.

## Materials and Methods

### Materials

In the present study, *Morchella* strain MB1 was isolated from‍ ‍alga-associated *Morchella* in moist soil on the campus of Jilin Normal University. *M. aeruginosa* used in this study was provided by the Institute of Hydrobiology, Chinese Academy of Sciences. The standard algal toxin products, MC-LR and MC-RR, were obtained from Beijing Biorebo Technology.

### Isolation and identification of *Morchella* strain MB1

Purified strain MB1 was inoculated on Potato Dextrose Agar (PDA) culture medium for 3–20‍ ‍d, and colony morphology was observed. A fungal genomic DNA extraction kit (Beijing Solaibao Technology) was used to extract total DNA from samples. The Internal Transcribed Spacer (ITS) region is a non-coding DNA sequence located between the 18S and 28S rRNA genes within the fungal genome. This sequence is used as a mole­cular marker for fungal identification and phylogenetic ana­lyses (Tretter *et al.*, 2013; Ao *et al.*, 2019). The ITS of the nuclear rRNA genes fragment of samples was amplified and sequenced using the universal primers ITS1 (5′-TCCGTAGGTGAACCTGC-3′) and ITS4 (5′-TCCTCCGCTTATTGATATGCG-3′) (Ramadurai and Balasundaram, 2020), and a phylogenetic tree was constructed by the maximum-likelihood method using MEGA7 software (Liu *et al.*, 2018a). Sequences generated in the present study were deposited in GenBank under the accession number PP256506.

### Inhibitory activity of strain MB1 against *M. aeruginosa*

*M. aeruginosa* was cultured in 250‍ ‍mL of BG11 liquid medium (Wang *et al.*, 2013a; Kucala *et al.*, 2021) and incubated under a 12-h dark/light cycle at 25°C with a light intensity of 500‍ ‍μmol m^–2^ s^–1^ for a duration of 15‍ ‍d (Chen *et al.*, 2014; Omidi *et al.*, 2019). After‍ ‍collecting *M. aeruginosa* in the logarithmic growth phase, the concentration of the algal solution was adjusted to 2–2.5×10^7^‍ ‍cells‍ ‍mL^–1^ as the initial concentration. The control group contained an inactivated *M. aeruginosa* solution (CK1) and active *M. aeruginosa* solution (CK2). Stationary phase strain MB1 was added to both CK1 and CK2. We collected 250‍ ‍mL of the *M. aeruginosa* solution and co-cultured it with dry weights of 0, 0.2424, 0.4848, 0.7272, 0.9696, 1.2120, and 1.4544‍ ‍g MB1, designated as M0, M1, M2, M3, M4, M5, and M6, respectively. The experiment was repeated three times for each group on days 0, 1, 2, 3, and 4, respectively. The solution was aerated using an air pump to maintain the activity of strain MB1.

### Assessment of the removal of nitrogen and phosphorus

The *M. aeruginosa* solution was co-cultured with strain MB1 as previously reported (Cao *et al.*, 2021). Following treatment, the culture medium was collected on days 0, 1, 2, and 3. Nitrogen and phosphorus contents in the culture medium were detected using a high-precision COD multiparameter tester (HI83099; HANNA Instruments Model) according to the manufacturer’s instructions. This experiment was repeated three times, and nitrogen and phosphorus removal rates were calculated using the following formula:

removal rate=(initial concentration–treated concentration)×100%/initial concentration

### Assessment of the ability of strain MB1 to remove MC-LR and MC-RR

*M. aeruginosa* cultured for 15‍ ‍d was frozen at –20°C for 24‍ ‍h and then dissolved by heating in a water bath at 50°C, followed by ultrasonication for 30‍ ‍min. The algal liquid was then centrifuged at 5,000‍ ‍rpm at 4°C for 20‍ ‍min, and the resulting supernatant was collected as MC MC-LR and MC-RR samples. *Morchella* strain MB1, represented as M0, M1, M2, M3, M4, M5, and M6, was individually introduced into an MC solution for a 3-d co-cultivation period. HPLC was used to detect the contents of MC-LR and MC-RR. A total of 0.9696‍ ‍g of *Morchella* was then inoculated into the MC solution, and the MC content was measured over a 5-d co-cultivation with strain MB1. This procedure was repeated in triplicate, and continuous aeration was ensured using an air pump to maintain the activity of strain MB1.

### Statistical ana­lysis

Statistical software SPSS 20.0 (IBM SPSS) was used to analyze the significance of differences in the present study. *P*<0.05 was considered to be significant.

## Results

### Identification of strain MB1

**Fig. 1.**
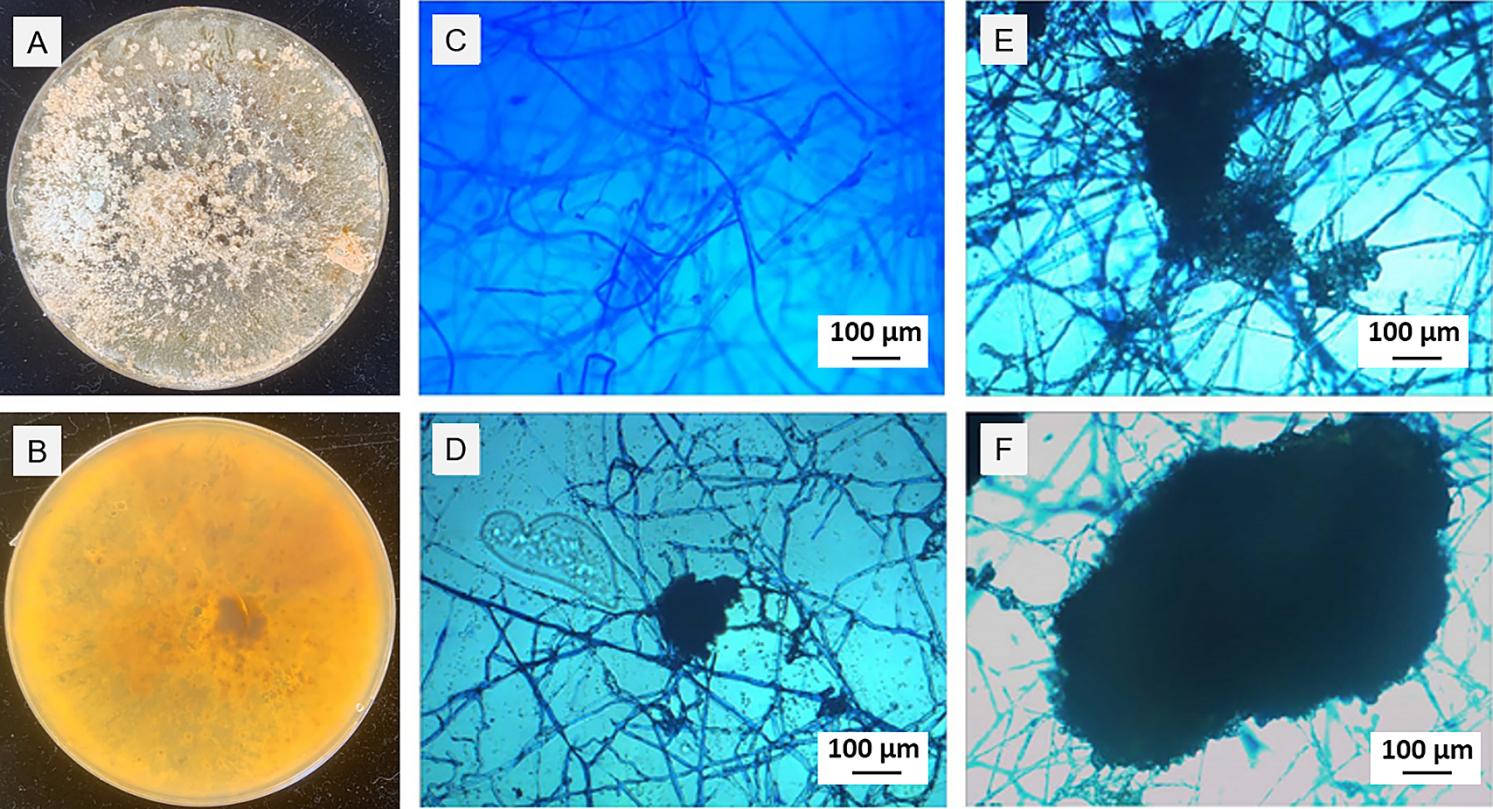
Morphological characteristics of strain MB1. (A) Strain MB1 was cultured at 25°C on PDA medium for 20 d. (B) Older mycelia in the center of the medium secreted brown pigment. (C) The mycelia of strain MB1 cultured for 20 d. (D, E, and F) Sclerotia of strain MB1 cultured for 20 d.

Strain MB1 was cultured on PDA to observe colony and mycelial morphologies. After an incubation at 25°C for 20‍ ‍d, mycelia gradually transitioned from transparent to light yellow, with some turning dark brown (Fig. 1A). The central older mycelia secreted a brown pigment that caused the discoloration of the medium (Fig. 1B), while maintaining a smooth surface. Thick mycelia exhibited numerous interlaced branches ranging from 1.5–13.5‍ ‍μm in diameter (Fig. 1C). The sclerotium consisted of closely packed mycelia of various shapes and sizes, lacking an evident epidermal structure, leading to the presumption that these sclerotia were microsclerotia (Fig. 1D, E, and F).

**Fig. 2.**
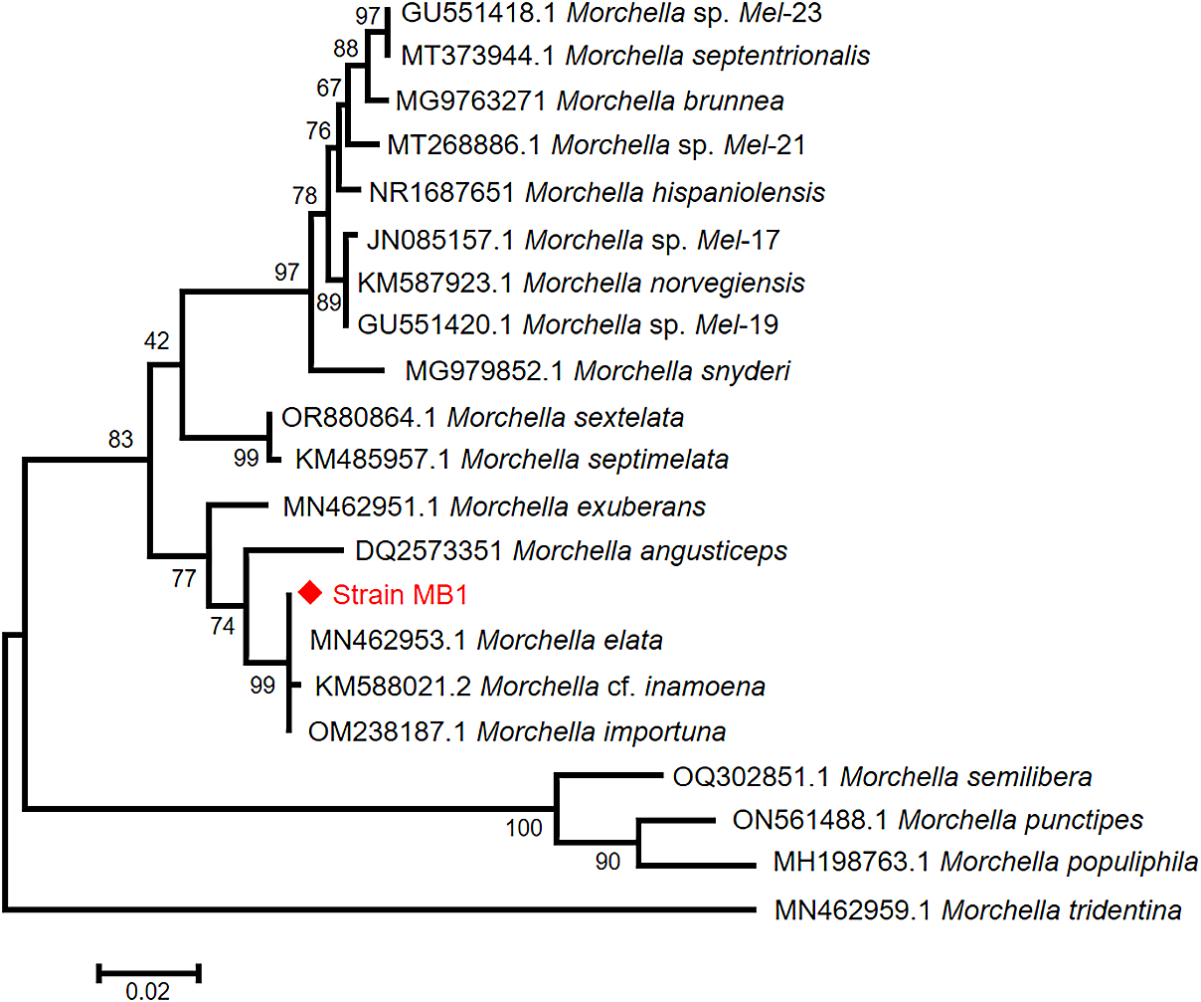
Maximum-likelihood phylogenetic tree generated from sequences of the nuc rRNA genes ITS region.

To analyze the phylogenetic relationship of MB1 within *Morchella*, a phylogenetic tree was constructed using ITS gene sequences. All analyzed sequences were classified into three groups (Fig. 2), which was consistent with previous studies (Richard *et al.*, 2015), with MB1 and *M. elata* placed in the same clade, indicating a close relationship, and MB1 isolated from *M. semilibera* and *M. tridentina*.

### Inhibitory effects of MB1 against *M. aeruginosa*

**Fig. 3.**
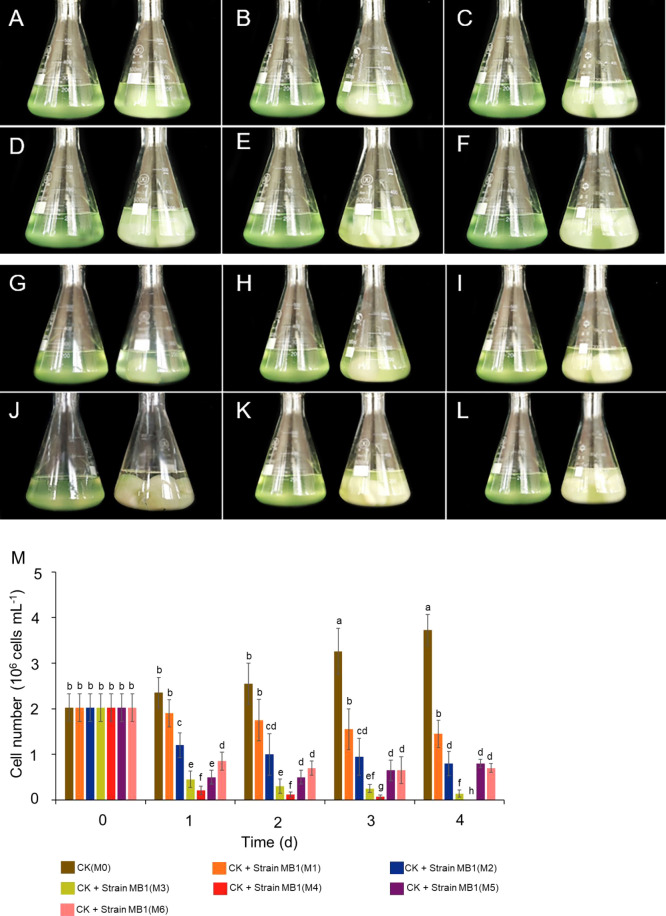
Co-cultivation of *Microcystis aeruginosa* with *Morchella esculenta* for 4 d. The *M. aeruginosa* solution co-cultured with strain MB1 on day 0 (A, B, C, D, E, and F) and on day 1 (G, H, I, J, K, and L). A, B, C, D, E, F, G, H, I, J, K, and L (left) inoculated with M0 strain MB1. A (right) inoculated with M1 strain MB1. B (right) inoculated with M2 strain MB1. C (right) inoculated with M3 strain MB1. D (right) inoculated with M4 strain MB1. E (right) inoculated with M5 strain MB1. F (right) inoculated with M6 strain MB1. G, H, I, J, K, and L (left) inoculated with M0 strain MB1. G (right) inoculated with M1 strain MB1. H (right) inoculated with M2 strain MB1. I (right) inoculated with M3 strain MB1. J (right) inoculated with M4 strain MB1. K (right) inoculated with M5 strain MB1. L (right) inoculated with M6 strain MB1. (M) The cell numbers of *M. aeruginosa* in co-cultured solutions with M0, M1, M2, M3, M4, M5, and M6.

To investigate the inhibitory effects of *Morchella* on the growth of *M. aeruginosa*, we co-cultured strain MB1 in the stationary phase and *M. aeruginosa* in the logarithmic phase. Notably, the *M. aeruginosa* solution co-cultured with M2, M3, M5, and M6 exhibited gradual clarity, while the solution co-cultivated with M4 became almost clear on day 1 (Fig. 3A, B, C, D, E, F, G, H, I, J, K, and L). The number of *M. aeruginosa* cells cultured with strain MB1 was calculated and the results obtained revealed significant reductions in M2, M3, M4, M5, and M6 on day 1 (Fig. 3M). *M. aeruginosa* cell counts significantly decreased in the M1, M2, M3, M4, M5, and M6 groups on days 2–4. Moreover, M4 resulted in zero *M. aeruginosa* cells on day 4 (Fig. 3M and S1). These results indicated that *Morchella* effectively inhibited the growth of *M. aeruginosa*.

### Removal of nitrogen and phosphorus by *Morchella* strain MB1

**Fig. 4.**
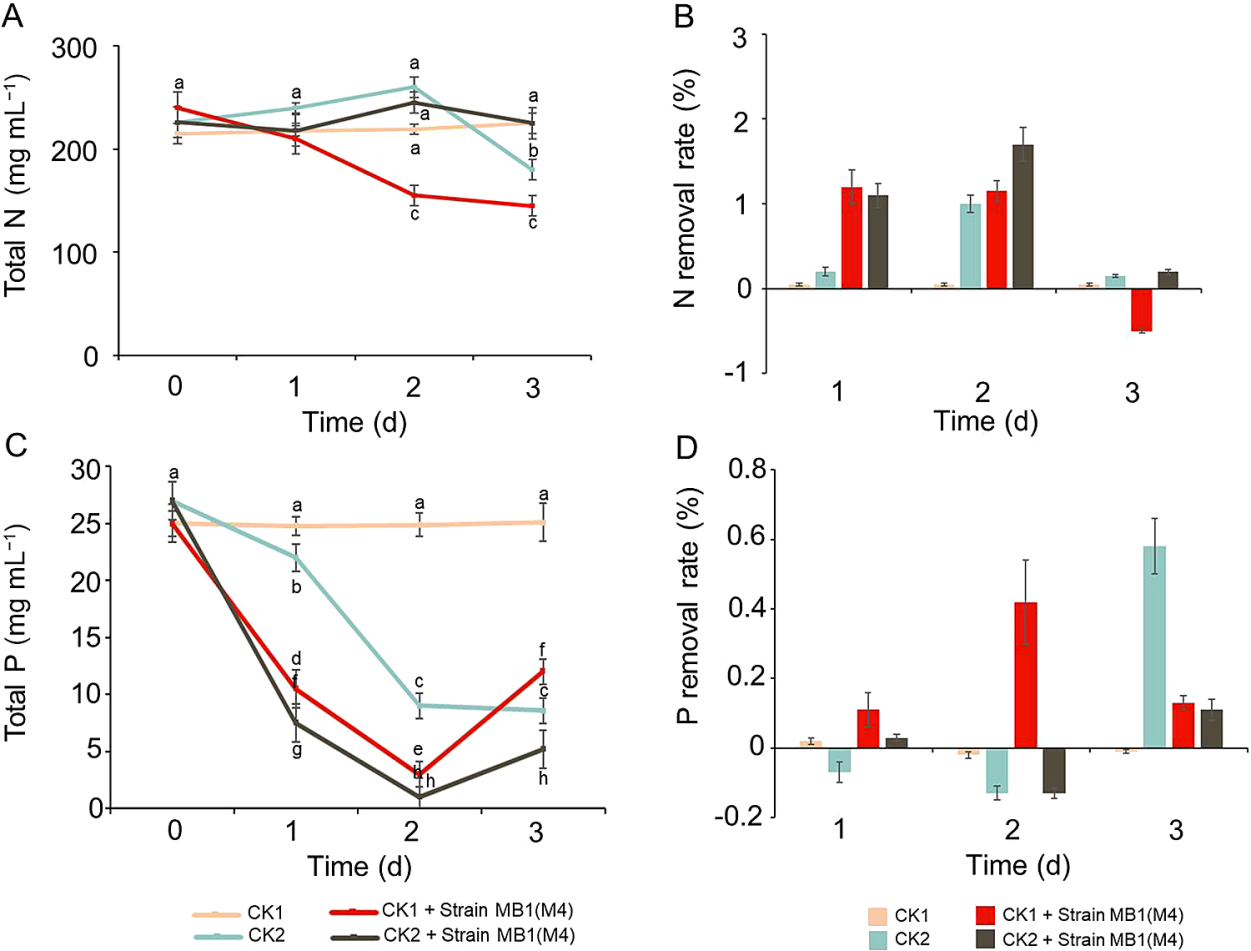
Removal of nitrogen and phosphorus from the *Microcystis aeruginosa* solution by strain MB1. (A) Total nitrogen content. (B) Nitrogen removal rate. (C) Total phosphorus content. (D) Phosphorus removal rate. CK1: inactive *M. aeruginosa* solution. CK2: active *M. aeruginosa* solution. CK1+strain MB1 (M4): 0.9696‍ ‍g of strain MB1 was inoculated into the inactive *M. aeruginosa* solution. CK2+strain MB1 (M4): 0.9696‍ ‍g of strain MB1 was inoculated into the *M. aeruginosa* solution.

To investigate the capability of *Morchella* to remove nitrogen and phosphorus from the *M. aeruginosa* solution, two control groups were established: CK1 and CK2. M4 was inoculated into CK1 and CK2, and nitrogen and phosphorus concentrations in the solutions were measured. The nitrogen content in the inactivated *M. aeruginosa* solution significantly decreased 2‍ ‍d after the inoculation (CK2+strain MB1 vs. CK2). Additionally, the nitrogen content in the *M. aeruginosa* solution was significantly lower 1–2‍ ‍d after the inoculation with the CK1+strain MB1 than that with CK1 (Fig. 4A).

Following the inoculation, the phosphorus content sig­nificantly decreased in CK1+strain MB1 vs. CK1 and CK2+strain MB1 vs. CK2 (Fig. 4C). Furthermore, the removal ratios of nitrogen and phosphorus revealed that the nitrogen clearance rate significantly increased on days 1 and 2 after the inoculation with the inactive *M. aeruginosa* and active *M. aeruginosa* solutions (Fig. 4B), whereas the phosphorus clearance rate significantly increased on days 1 and 2 after the *M. aeruginosa* inoculation. (Fig. 4D). These results indicate that MB1 removed nitrogen and phosphorus from the *M. aeruginosa* solution.

### Removal of MCs by strain MB1

**Fig. 5.**
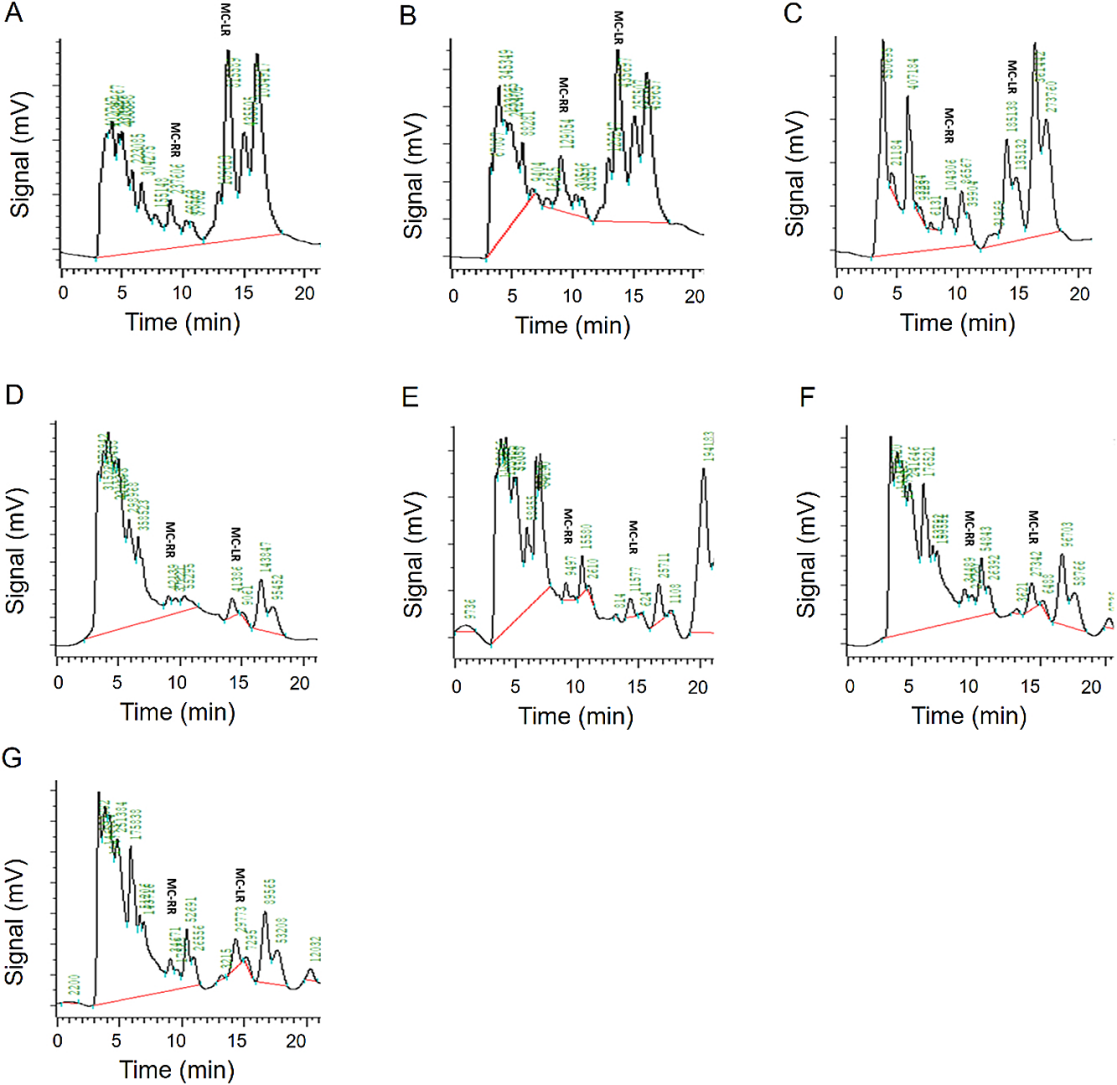
HPLC-based component and content ana­lyses with MB1 inoculated into MC solution. Component and content ana­lyses of different quantities of MB1, M0 (A), M1 (B), M2 (C), M3 (D), M4 (E), M5 (F), and M6 (G).

**Fig. 6.**
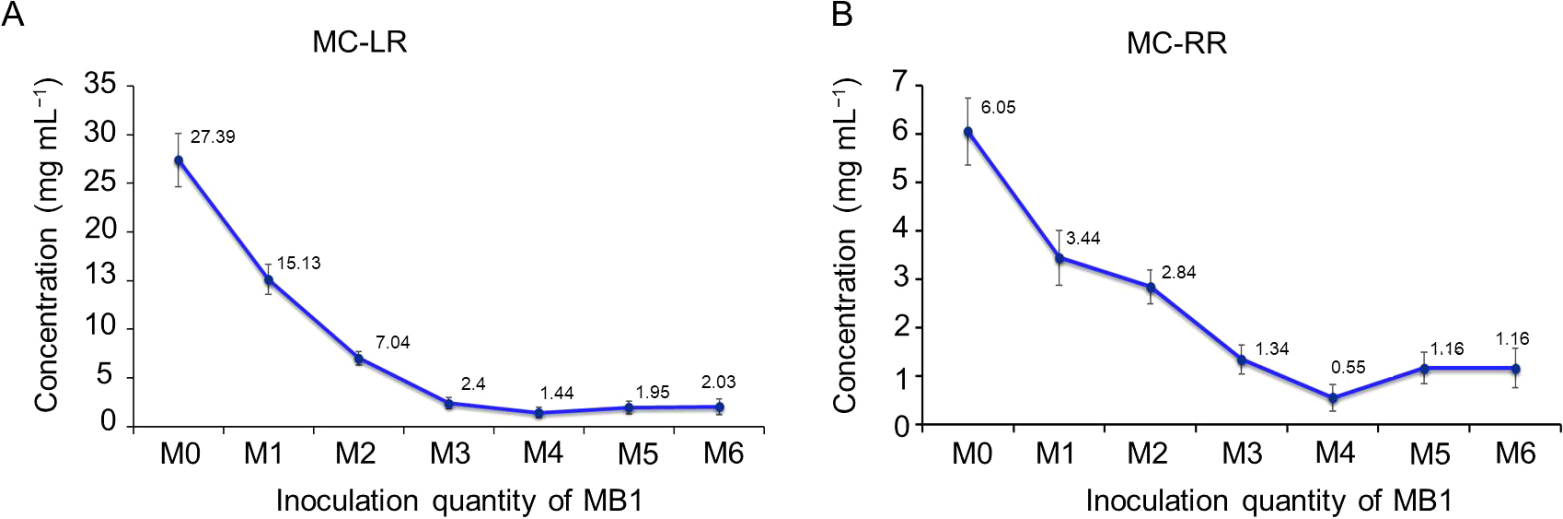
Removal of MC-LR and MC-RR by strain MB1. (A) MC-LR content after the inoculation with M0 to M6 for 5 d. (B) MC-RR content after the inoculation with M0 to M6 for 5 d.

To investigate the ability of strain MB1 to remove MCs from *M. aeruginosa*, it was introduced into the MC solution. In M0, peak areas for MC-LR were measured at 815,589, while MC-RR registered at 237,606 (Fig. 5A). Across various volumes of the MB1 inoculation (M1 to M6), a consistent decrease in peak areas for both MC-LR and MC-RR was observed against M0 (Fig. 5A, B, C, D, E, F, and G). The minimum peak area of MC-LR occurred with M4, measuring 11,577. Similarly, M4 exhibited the largest decrease in the MC-RR peak area, registering at 9,497 (Fig. 5E). Furthermore, we analyzed the contents of MC-LR and MC-RR in the MC solution. The initial concentrations of MC-LR and MC-RR, 27.39 and 6.05‍ ‍μg mL^–1^, respectively, significantly decreased with varying amounts of the MB1 inoculation (M1–M6) into the solution. Notably, the lowest concentrations of MC-LR and MC-RR in the MC solution were achieved with M4 (Fig. 6A and B). Furthermore, M4 exhibited the most effective removal of MC-LR and MC-RR on day 5 after the inoculation (Fig. S2). These results suggest that strain MB1 removed MCs. The potential of *Morchella* to enhance water quality by reducing nitrogen and phosphorus levels and removing MCs positions it as a valuable asset for wastewater treatment.

## Discussion

The majority of biological methods use bacteria and fungi to inhibit the growth of algae and destroy MC structures (Bouaicha *et al.*, 2019). Algae-lyzing microorganisms, including fungi, including *Trichoderma* and *Penicillium* (Mohamed *et al.*, 2014; Han *et al.*, 2021), bacteria, such as *Pseudomonas* (Wang *et al.*, 2013b; Liu *et al.*, 2014), *Bacillus* (Shao *et al.*, 2013), and *Streptomyces* sp. (Kong *et al.*, 2014), as well as MC-degrading *Acinetobacter* (Yi *et al.*, 2015), have demonstrated the ability to inhibit the growth of *M. aeruginosa* and even remove MCs. However, it may take up to 8‍ ‍d for these algal-bacteria to dissolve algae, achieving a removal rate of approximately 90% (Kong *et al.*, 2014). In contrast, *Morchella* strain MB1 used in the present study exhibited a removal rate of approximately 100% against *M. aeruginosa* cells within just 4 d. This result suggests that *Morchella* exhibits superior efficiency and efficacy for the removal of *M. aeruginosa* to those of algae-lytic bacteria.

*Morchella* is a common edible fungus that generally thrives in challenging environments characterized by low temperatures, darkness, and humidity. It is also known for its richness in polysaccharides (Zhang *et al.*, 2023), and previous studies showed that these microorganisms produced polysaccharides in the stationary phase (Xu *et al.*, 2008; Qian *et al.*, 2023). However, the precise mechanisms underlying the effects of *Morchella* on MCs, whether it involves degradation or polysaccharide adsorption, remain unclear. *Morchella* may use nitrogen and phosphorus in water as nutrients for its growth, indicating its consumption of nitrogen and phosphorus in the *M. aeruginosa* solution used in this experiment. *Morchella* exhibits robust vitality and has the capability to form sclerotia in order to sustain growth in nutrient-deprived environments without under­going natural apoptosis (Liu *et al.*, 2018b; Sun *et al.*, 2020). Moreover, it does not produce spores that may lead to secondary pollution, making it an optimal choice for managing *M. aeruginosa*. However, due to its classification as a soil‍ ‍fungus, the survival and reproduction of *Morchella* are‍ ‍limited in the natural aquatic environment where *M. aeruginosa*, a producer of MC toxins, thrives.

The potential of *Morchella* to enhance water quality by reducing nitrogen and phosphorus levels and removing MCs positions it as a valuable asset for wastewater treatment. Therefore, *Morchella* has potential as a promising biological technique to remove *Microcystis* in a short time and even MCs, thereby safeguarding both the environment and human well-being. Nevertheless, further investigations are needed to establish whether *Morchella* inhibits MC biosynthesis as well as the underlying mechanisms. Additionally, it is important to elucidate the specific mechanisms responsible for the inhibitory effects of *Morchella* on the growth and reproduction of *M. aeruginosa*.

## Citation

Meng, X., Ban, M., Wu, Z., Huang, L., Wang, Z., and Cheng, Y. (2024) *Morchella* Effectively Removes Microcystins Produced by *Microcystis aeruginosa*. *Microbes Environ ***39**: ME23101.

https://doi.org/10.1264/jsme2.ME23101

## Supplementary Material

Supplementary Material

## References

[B1] Alosman, M., Cao, L., Massey, I.Y., and Yang, F. (2021) The lethal effects and determinants of microcystin-LR on heart: a mini review. Toxin Rev 40: 517–526.

[B2] Ao, T., Deb, C.R., and Rao, S.R. (2019) Molecular strategies for identification and characterization of some wild edible mushrooms of Nagaland, India. Mol Biol Rep 47: 621–630. 31754929 10.1007/s11033-019-05170-2

[B3] Bouaicha, N., Miles, C.O., Beach, D.G., Labidi, Z., Djabri, A., Benayache, N.Y., et al. (2019) Structural diversity, characterization and toxicology of microcystins. Toxins (Basel) 11: 714. 31817927 10.3390/toxins11120714 PMC6950048

[B4] Campos, A., and Vasconcelos, V. (2010) Molecular mechanisms of microcystin toxicity in animal cells. Int J Mol Sci 11: 268–287. 20162015 10.3390/ijms11010268 PMC2821003

[B5] Cao, L., Huang, F., Massey, I.Y., Wen, C., Zheng, S., Xu, S., et al. (2019) Effects of microcystin-LR on the microstructure and inflammation-related factors of jejunum in mice. Toxins (Basel) 11: 428. 31438657 10.3390/toxins11090482 PMC6783826

[B6] Cao, S., Zhang, D., Teng, F., Liao, R., Cai, Z., Tao, Y., et al. (2021) Inhibitory effects of ultralow-dose sodium hypochlorite on *Microcystis aeruginosa* and *Chlorella vulgaris*: Differences in sensitivity and physiology. Sci Total Environ 774: 145638.

[B7] Chen, H.W., Huang, W.J., Wu, T.H., and Hon, C.L. (2014) Effects of extracellular polymeric substances on the bioaccumulation of mercury and its toxicity toward the cyanobacterium *Microcystis aeruginosa*. J Environ Sci Heal A 49: 1370–1379. 10.1080/10934529.2014.928249 25072768

[B8] de la Cruz, A.A., Antoniou, M.G., Hiskia, A., Pelaez, M., Song, W., O’Shea, K.E., et al. (2011) Can we effectively degrade microcystins?—Implications on human health. Anti-Cancer Agents Med Chem 11: 19–37. 10.2174/187152011794941217 21269255

[B9] Drobac, D., Tokodi, N., Simeunović, J., Baltić, V., Stanić, D., and Svirćev, Z. (2013) Human exposure to cyanotoxins and their effects on health. Arh Hig Rada Toksikol 64: 119–130. 23819940 10.2478/10004-1254-64-2013-2320

[B10] Du, X., Liu, H., Yuan, L., Wang, Y., Ma, Y., Wang, R., et al. (2019) The diversity of cyanobacterial toxins on structural characterization, distribution and identification: A systematic review. Toxins (Basel) 11: 530. 31547379 10.3390/toxins11090530 PMC6784007

[B11] Duan, X., Sanan, T., de la Cruz, A., He, X., Kong, M., and Dionysiou, D.D. (2018) Susceptibility of the algal toxin microcystin-LR to UV/chlorine process: Comparison with chlorination. Environ Sci Technol 52: 8252–8262. 29920077 10.1021/acs.est.8b00034 PMC7382943

[B12] Dziga, D., Wasylewski, M., Wladyka, B., Nybom, S., and Meriluoto, J. (2013) Microbial degradation of microcystins. Chem Res Toxicol 26: 841–852. 23621464 10.1021/tx4000045

[B13] Han, S., Zhou, Q., Lilje, O., Xu, W., Zhu, Y., and van Ogtrop, F.F. (2021) Inhibition mechanism of *Penicillium chrysogenum* on *Microcystis aeruginosa* in aquaculture water. J Clean Prod 299: 126829.

[B14] He, Q., Wang, W., Xu, Q., Liu, Z., Teng, J., Yan, H., et al. (2022) Microcystins in water: Detection, microbial degradation strategies, and mechanisms. Int J Environ Res Public Health 19: 13175. 36293755 10.3390/ijerph192013175 PMC9603262

[B15] Hu, X., Hu, X., Tang, C., Wen, S., Wu, X., Long, J., et al. (2017) Mechanisms underlying degradation pathways of microcystin-LR with doped TiO2 photocatalysis. Chem Eng J 330: 355–371.

[B16] Huo, D., Gan, N., Geng, R., Cao, Q., Song, L., Yu, G., et al. (2021) Cyanobacterial blooms in China: diversity, distribution, and cyanotoxins. Harmful Algae 109: 102106. 34815019 10.1016/j.hal.2021.102106

[B17] Kong, Y., Zhu, L., Zou, P., Qi, J., Yang, Q., Song, L., et al. (2014) Isolation and characterization of dissolved organic matter fractions from antialgal products of *Microcystis aeruginosa*. Environ Sci Pollut Res 21: 3946–3954. 10.1007/s11356-013-2114-y 24293343

[B18] Kucala, M., Saladyga, M., and Kaminski, A. (2021) Phytoremediation of CYN, MC-LR and ANTX-a from water by the submerged macrophyte *Lemna trisulca*. Cells 10: 699. 33801135 10.3390/cells10030699 PMC8004190

[B19] Lee, J., Lee, S., and Jiang, X. (2017) Cyanobacterial toxins in freshwater and food: Important sources of exposure to humans. Annu Rev Food Sci Technol 8: 281–304. 28245155 10.1146/annurev-food-030216-030116

[B20] Li, J., Li, R., and Li, J. (2017) Current research scenario for microcystins biodegradation—A review on fundamental knowledge, application prospects and challenges. Sci Total Environ 595: 615–632. 28407581 10.1016/j.scitotenv.2017.03.285

[B21] Liu, J.L., Zhao, M., Zhang, W.Q., and Huang, M.Y. (2018a) Expansion of the IL1B gene family in the pig. Proc Natl Acad Sci U S A 115: E5843–E5844. 29880716 10.1073/pnas.1806496115 PMC6042090

[B22] Liu, Q., Ma, H., Zhang, Y., and Dong, C. (2018b) Artificial cultivation of true morels: current state, issues and perspectives. Crit Rev Biotechnol 38: 259–271. 28585444 10.1080/07388551.2017.1333082

[B23] Liu, Z.Z., Zhu, J.P., Li, M., Xue, Q.Q., Zeng, Y., and Wang, Z.P. (2014) Effects of freshwater bacterial siderophore on *Microcystis* and *Anabaena*. Biol Control 78: 42–48.

[B24] Massey, I.Y., Yang, F., Ding, Z., Yang, S., Guo, J., Tezi, C., et al. (2018) Exposure routes and health effects of microcystins on animals and humans: A mini-review. Toxicon 151: 156–162. 30003917 10.1016/j.toxicon.2018.07.010

[B25] Massey, I.Y., and Yang, F. (2020) A mini review on microcystins and bacterial degradation. Toxins (Basel) 12: 268. 32326338 10.3390/toxins12040268 PMC7232508

[B26] Mohamed, Z.A., Hashem, M., and Alamri, S.A. (2014) Growth inhibition of the cyanobacterium *Microcystis aeruginosa* and degradation of its microcystin toxins by the fungus *Trichoderma citrinoviride*. Toxicon 86: 51–58. 24874888 10.1016/j.toxicon.2014.05.008

[B27] Neilan, B.A., Pearson, L.A., Muenchhoff, J., Moffitt, M.C., and Dittmann, E. (2012) Environmental conditions that influence toxin biosynthesis in cyanobacteria. Environ Microbiol 15: 1239–1253. 22429476 10.1111/j.1462-2920.2012.02729.x

[B28] Omidi, A., Esterhuizen-Londt, M., and Pflugmacher, S. (2019) Interspecies interactions between *Microcystis aeruginosa* PCC 7806 and *Desmodesmus subspicatus* SAG 86.81 in a co-cultivation system at various growth phases. Environ Int 131: 105052. 31357091 10.1016/j.envint.2019.105052

[B29] Qian, L., Du, M., Yang, X., Wang, Q., Huang, S., Ma, Y., et al. (2023) Microana­lysis characterization and immunomodulatory effect for selenium-enriched polysaccharide from *Morchella esculenta* (L.) Pers. Molecules 28: 2885. 37049647 10.3390/molecules28072885 PMC10096435

[B30] Ramadurai, S., and Balasundaram, U. (2020) Rhizomicrobiomics of Caesalpinia bonducella, a wonder plant for PCOS treatment. Physiol Mol Biol Plants 26: 2453–2463. 33424158 10.1007/s12298-020-00915-x PMC7772120

[B31] Richard, F., Bellanger, J.M., Clowez, P., Hansen, K., O’Donnell, K., Urban, A., et al. (2015) True morels (*Morchella*, Pezizales) of Europe and North America: evolutionary relationships inferred from multilocus data and a unified taxonomy. Mycologia 107: 359–382. 25550303 10.3852/14-166

[B32] Shao, J., Jiang, Y., Wang, Z., Peng, L., Luo, S., Gu, J., et al. (2013) Interactions between algicidal bacteria and the cyanobacterium *Microcystis aeruginosa*: lytic characteristics and physiological responses in the cyanobacteria. Int J Environ Sci Te 11: 469–476.

[B33] Sun, X., Liu, D., Wang, Y., and Ma, A. (2020) Biogenesis of macrofungal sclerotia: influencing factors and mole­cular mechanisms. Appl Microbiol Biotechnol 104: 4227–4234. 32198573 10.1007/s00253-020-10545-8

[B34] Teng, J., Song, M., Xu, Q., Zou, Q., Zhang, H., Yin, C., et al. (2023) Purification and activity of the second recombinant enzyme for biodegrading linearized microcystins by *Sphingopyxis* sp. USTB-05. Toxins (Basel) 15: 494. 37624251 10.3390/toxins15080494 PMC10467064

[B35] Tretter, E.D., Johnson, E.M., Wang, Y., Kandel, P., and White, M.M. (2013) Examining new phylogenetic markers to uncover the evolutionary history of early-diverging fungi: comparing MCM7, TSR1 and rRNA genes for single- and multi-gene ana­lyses of the *Kickxellomycotina*. Persoonia 30: 106–125. 24027350 10.3767/003158513X666394 PMC3734964

[B36] Wang, X., Xie, M., Wu, W., Shi, L., Luo, L., and Li, P. (2013b) Differential sensitivity of colonial and unicellular *Microcystis* strains to an algicidal bacterium *Pseudomonas aeruginosa*. J Plankton Res 35: 1172–1176.

[B37] Wang, Z., Luo, Z., and Yan, C. (2013a) Accumulation, transformation, and release of inorganic arsenic by the freshwater cyanobacterium *Microcystis aeruginosa*. Environ Sci Pollut Res 20: 7286–7295. 10.1007/s11356-013-1741-7 23636594

[B38] Woolbright, B.L., Williams, C.D., Ni, H., Kumer, S.C., Schmitt, T., Kane, B., et al. (2017) Microcystin-LR induced liver injury in mice and in primary human hepatocytes is caused by oncotic necrosis. Toxicon 125: 99–109. 27889601 10.1016/j.toxicon.2016.11.254 PMC5193107

[B39] Xu, H., Sun, L.-P., Shi, Y.-Z., Wu, Y.-H., Zhang, B., and Zhao, D.-Q. (2008) Optimization of cultivation conditions for extracellular polysaccharide and mycelium biomass by *Morchella esculenta* As51620. Biochem Eng J 39: 66–73.

[B40] Yi, Y.L., Yu, X.B., Zhang, C., and Wang, G.X. (2015) Growth inhibition and microcystin degradation effects of *Acinetobacter guillouiae* A2 on *Microcystis aeruginosa*. Res Microbiol 166: 93–101. 25638018 10.1016/j.resmic.2014.12.013

[B41] Zhan, M.-m., and Hong, Y. (2022) Recent advances in technologies for removal of microcystins in water: a review. Curr Pollut Rep 8: 113–127.

[B42] Zhang, J., Zhao, J., Liu, G., Li, Y., Liang, L., Liu, X., et al. (2023) Advance in *Morchella* sp. polysaccharides: Isolation, structural characterization and structure-activity relationship: A review. Int J Biol Macromol 247: 125819. 37455001 10.1016/j.ijbiomac.2023.125819

[B43] Zhu, P., Chen, G., Liu, Y., Wang, Q., Wang, M., and Hu, T. (2022) Microcystin-leucine arginine exhibits adverse effects on human aortic vascular smooth muscle cells in vitro. Toxicol In Vitro 84: 105450. 35905885 10.1016/j.tiv.2022.105450

