# Supplementary material for: Morchella Effectively Removes Microcystins Produced by Microcystis aeruginosa
Source: Microbes Environ. 2024 May 18;39(2):ME23101. doi: 10.1264/jsme2.ME23101 (PMC11220450; doi:10.1264/jsme2.ME23101)
Supplement: Supplementary file 1 — Supplementary Material [file 39_23101_s1.pdf]

Morchella Effectively Removes Microcystins Produced by *Microcystis aeruginosa*

XinChao Meng[1], MeiHan Ban[1], ZhaoYang Wu[1], LiLong Huang[1], ZiCheng Wang[2], and YunQing Cheng [1*]

[1]*Jilin Provincial Key Laboratory of Plant Resource Science and Green Production, Jilin Normal University, Siping* 136000*, China; and* [2]*Department of Microbiology, Oregon State University, Corvallis, OR 97331, United States*

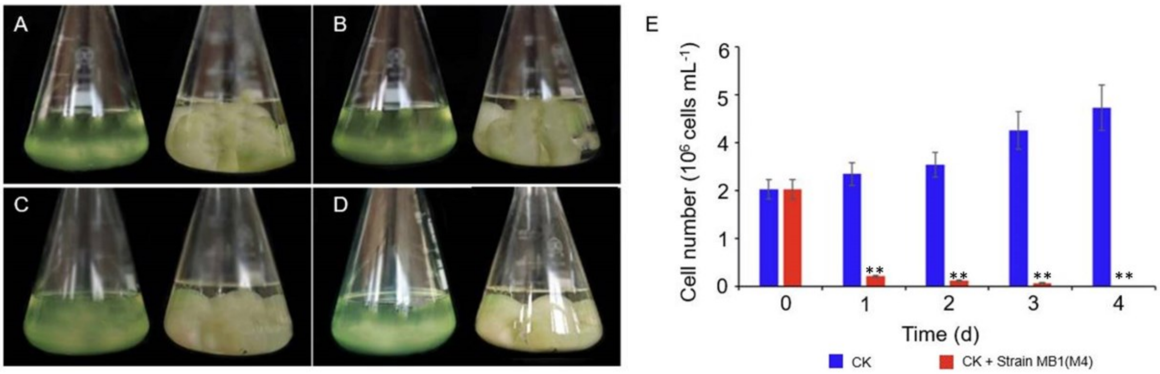

Fig. S1 Co-cultivation of *M. aeruginosa* with MB1 for 4 d

A–D (left) inoculated with M0 for 1–4 d, respectively. A–D (right) inoculated with M4 for 1–4 d, respectively. (E) The cell number of *M. aeruginosa* in co-cultured solutions with M4 for 0–4 d. ** represent $p < 0.01$.

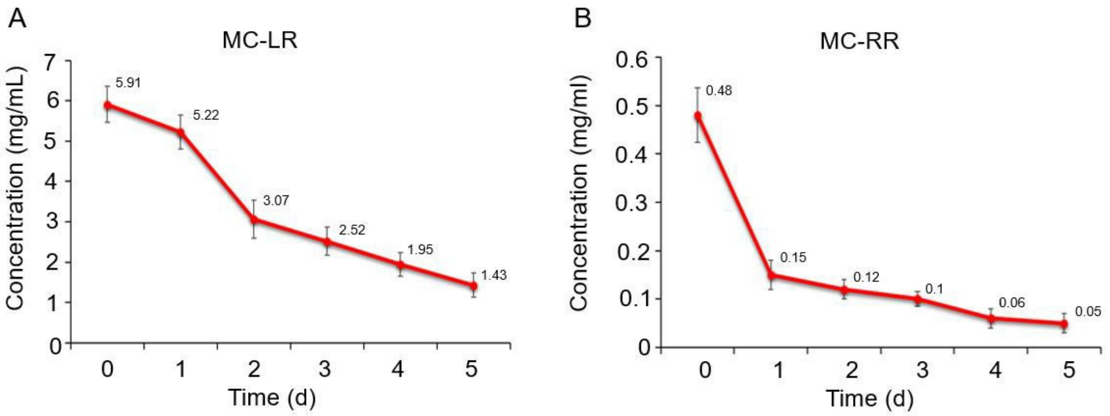

Fig. S2 Removal of MC-LR and MC-RR by Strain MB1

(A) MC-LR content after inoculation with M4 for 0-5 d. (B) MC-RR content after inoculation with M4 for 0-5 d.
